# Efflux Pump Inhibition and Resistance Modulation in *Mycobacterium smegmatis* by *Peucedanum ostruthium* and Its Coumarins

**DOI:** 10.3390/antibiotics10091075

**Published:** 2021-09-05

**Authors:** Katarina Šimunović, Julia Solnier, Fabian Alperth, Olaf Kunert, Sonja Smole Možina, Franz Bucar

**Affiliations:** 1 Department of Pharmacognosy, Institute of Pharmaceutical Sciences, University of Graz, Beethovenstraße 8, 8010 Graz, Austria; katarina.simunovic@bf.uni-lj.si (K.Š.); julia.solnier@uni-graz.at (J.S.); fabian.alperth@uni-graz.at (F.A.); 2 Department of Food Science and Technology, Biotechnical Faculty, University of Ljubljana, Jamnikarjeva 101, 1000 Ljubljana, Slovenia; sonja.smole-mozina@bf.uni-lj.si; 3 Department of Pharmaceutical Chemistry, Institute of Pharmaceutical Sciences, University of Graz, Beethovenstraße 8, 8010 Graz, Austria; olaf.kunert@uni-graz.at

**Keywords:** *Mycobacterium smegmatis*, efflux pump inhibitor, *Peucedanum ostruthium*, antibacterial, ostruthin, imperatorin, ethidium bromide accumulation, resistance modulation

## Abstract

Antibiotic resistance is a growing problem and may become the next major global health crisis if no timely actions are taken. Mycobacterial infections are widespread and, due to antibiotic resistance, also hard to treat and a major cause of mortality. Natural compounds have the potential to increase antibiotic effectiveness due to their resistance modulatory and antimicrobial effects. In this study, *Peucedanum ostruthium* extracts, fractions, and isolated compounds were investigated regarding their antimicrobial and resistance-modulatory effects as well as efflux pump inhibition in *Mycobacterium smegmatis.* *P. ostruthium* extracts were found to have anti-mycobacterial potential and resistance modulating effects on ethidium bromide activity. The major antibacterial effect was attributed to ostruthin, and we found that the more lipophilic the substrate, the greater the antimicrobial effect. Imperatorin caused potent modulatory effects by interfering with the action of the major LfrA efflux pump in *M. smegmatis*. The plant *P. ostruthuim* has a complex effect on *M. smegmatis,* including antibacterial, efflux pump inhibition, resistance modulation, and membrane permeabilization, and its major constituents, ostruthin and imperatorin, have a distinct role in these effects. This makes *P. ostruthium* and its coumarins promising therapeutics to consider in the fight against drug-resistant mycobacteria.

## 1. Introduction

Antibiotic resistance in bacteria is an increasing problem and, if ignored, it could have devastating consequences for humanity. It is estimated that about 700,000 people die each year of infections with resistant bacteria, and almost 1/3 of those are due to tuberculosis alone [1]. It is thus not surprising that the interest in multidrug- and extensively drug-resistant (MDR-XDR) mycobacteria, resistant against numerous antimicrobial drugs, is attracting much attention in recent years as it has become a critical global health concern. In fact, the rising number of MDR strains, especially in mycobacteria, including the *Mycobacterium tuberculosis* complex, and fast-growing non-tuberculous mycobacterial (NTM) strains [2,3], highlights the pressing need of discovering new antibacterial agents.

Mycobacteria holding a unique cell wall structure composed of long-chain fatty acids such as C_60_ to C_90_ mycolic acids regularly pose intrinsic resistance to a variety of antimicrobial agents [4]. Besides the pathogenic mycobacteria, such as the *M. tuberculosis* complex, several opportunistic NTM species that occur as saprophytes and commensals in the environment [5]. Non-tuberculous mycobacteria are fast-growing, non-obligatory pathogenic organisms, which, however, can cause opportunistic infections in immunocompromised patients [6,7]. One of them, *Mycobacterium smegmatis,* is frequently used as a low pathogenic and rapidly growing model strain [8] in the anti-tubercular drug screening based on its genomic similarities and a correlating antibiotic susceptibility profile to that of *M. tuberculosis* [9,10]. In the study of Li et al. [4], it was demonstrated that several of the efflux pump genes, such as *lfrA* as well as *M. tuberculosis* homologs of Rv1145, Rv1146, Rv1877, Rv2846c (*efpA*), and Rv3065 (*mmr* and *emrE*), are present and expressed in the *M. smegmatis* strain mc^2^ 155. Further, it was shown that specifically, the LfrA pump of *M. smegmatis* contributes to intrinsic drug resistance of this organism as the deletion of the *lfrA* gene or *mmr* homolog rendered the mutant strain more susceptible to antibacterial drugs such as fluoroquinolones, ethidium bromide, and acriflavine [4]. In fact, the *M. smegmatis* LfrA pump, a major facilitator superfamily (MFS) efflux transporter, was the first multidrug efflux pump documented for mycobacteria [11].

Among the well-known resistance mechanisms, such as inactivation of drugs and alteration of targets [12], efflux pumps (EPs) are one of the key tools used by mycobacteria in order to combat chemotherapeutic drugs [10,13]. This makes them an important target in the fight against resistance in mycobacteria. The identification of novel efflux pump inhibitors (EPIs), especially from natural sources, could provide an innovative approach in tackling the resistance of mycobacteria as supporting therapeutics.

*Peucedanum ostruthium* (L.) Koch (syn. *Imperatoria ostruthium* L.), also known as masterwort, is a flowering plant from the family Apiaceae, growing in the Alps and other mountains across central and southern Europe, where it is used as a medicinal plant against various diseases. Plants belonging to the *Peucedanum* genus have been known for centuries for their antibacterial efficiency against different human pathogens [14]. Such antibacterial properties have been shown for *P. ostruthium* and its coumarins against a variety of Gram-negative and Gram-positive organisms, including *Staphylococcus aureus*, *Streptococcus mutans*, *Staphylococcus epidermidis*, *Pseudomonas aeruginosa*, *Escherichia coli*, *Klebsiella pneumoniae*, and others [15,16]. In folk medicine, *Peucedanum* species have a very long tradition as natural remedies applied for the treatment of inflammatory diseases [17]. The dried roots of *P.* *ostruthium* are being prepared as teas and bitters for gastrointestinal ailments, respiratory diseases, and fatigue, whereas the ethanol extract (Radix imperatoriae) is applied for the treatment of typhus, fever, or as a diuretic, and topically used to treat ulcers [18,19].

Coumarins, such as imperatorin, ostruthol, oxypeucedanin hydrate, oxypeucedanin, ostruthin, and osthole, are naturally plant-derived compounds with a benzopyrone moiety and are among the most distinctive secondary metabolites found in *P. ostruthium* species [19,20]. Several naturally derived coumarins have been reported to possess diverse biological activities, including anti-inflammatory, antioxidant [21], anticancer [22], antifungal, and antimicrobial [23] effects. Some experimental work has indicated that imperatorin [24] and osthole [25] may also act as EPIs in *S. aureus*. These facts make coumarins and plants containing them interesting not only as antimicrobials but also as potential resistance modulators and EPIs.

Ostruthin (6-geranyl-7-hydroxycoumarin) isolated from the roots of *P. ostruthium* showed a significant anti-mycobacterial potential against numerous species of rapidly growing mycobacteria such as *M. abscesus*, *M. aurum*, *M. fortuitum*, *M. phlei* and *M. smegmatis* [26]. In particular, dichloromethane extracts from the roots of *P. ostruthium* proved to be effective against *M. fortuitum,* responsible for causing respiratory system infections [14]. By analyzing the structure-activity relationship of those coumarins inhibiting the formation of acid-fastness in mycobacteria, the position of a hydroxy group at C-7 appeared to be highly relevant [26,27].

Since MDR and XDR in mycobacteria have been rising, this study served to investigate the impact of natural extracts and pure coumarins derived from *P. ostruthium* on *M. smegmatis* as novel antimicrobials with a focus on the EPI and resistance modulation effect for the effective treatment of mycobacterial infections such as tuberculosis. After the chemical analysis of *P. ostruthium* hexane and ethanol extracts and its fractions, pure compounds oxypeucedanin hydrate, imperatorin, ostruthin, and an unknown compound were isolated. The antimicrobial activity of extracts, fractions, and pure compounds was determined against *M. smegmatis* mc^2^ 155 wild type, and to determine the involvement of the LfrA efflux pump in *M. smegmatis* potential resistance against *P. ostruthium*, also against the *M. smegmatis* mc^2^ 155 Δ*lfrA* mutant lacking a functional LfrA efflux pump. Furthermore, the extracts were evaluated for resistance-modulatory, EPI, and membrane-permeabilizing activity.

## 2. Results

### 2.1. Chemical Analysis

**Figure 1:**
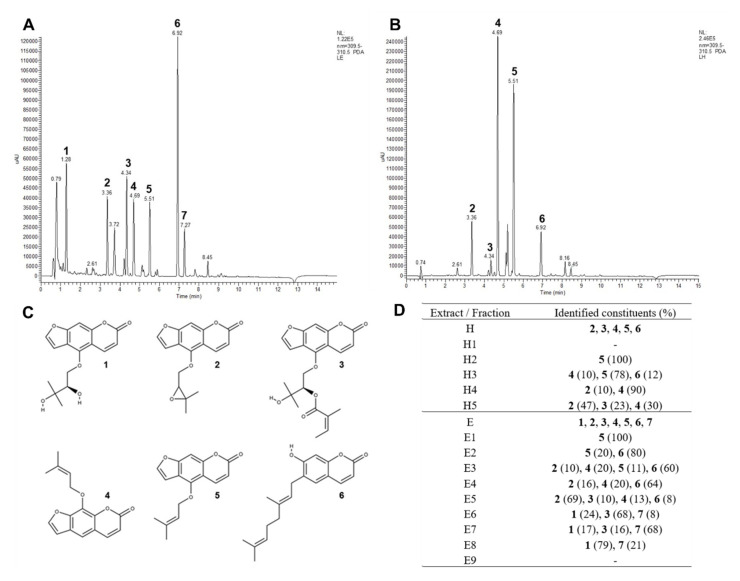
UV chromatogram of *P. ostruthium* ethanol (E) (**A**) and hexane (H) (**B**) extracts at 310 nm, molecular structures of main identified coumarins in *Peucedanum ostruthium* extracts oxypeucedanin hydrate (**1**), oxypeucedanin (**2**), ostruthol (**3**), imperatorin (**4**), isoimperatorin (**5**), ostruthin (**6**) (**C**) and constituents identified in extracts and subsequent fractions according to LC-PDA-ESI-MS^n^ results with the relative composition of identified coumarins in subfractions (%) (**D**).

Six constituents (Table 1 and Figure 1) were identified in *P. ostruthium* extracts according to literature references [19,28]. One additional compound could only be tentatively identified (Figure 1C). The compositions of fractions in alignment with results for raw ethanol (E, Figure 1A) and hexane (H, Figure 1B) extracts are shown in Figure 1D. For imperatorin (**4**) and isoimperatorin (**5**), the fragmentation of *m*/*z* 203 in MS^2^ is in alignment with loss of the prenyl group minus one proton accounting for −69 u in MS^1^. The molecular ion [M + H]^+^ with *m*/*z* 271 can be tracked with low intensity in the respective peaks in full MS (MS^1^). Compound **7** shows a molecular ion [M + H]^+^ of *m*/*z* 603 in MS^1^. UV maxima indicate a combination of two coumarin substructures, one being a simple coumarin such as ostruthin (**6**). The addition of molecular masses of ostruthin (298 g/mol) and oxypeucedanin hydrate (**1**) (304 g/mol) is in alignment with a molecular ion [M + H]^+^ of *m*/*z* 603 for the complex. The most prominent fragment ion in MS^2^ of 299 *m*/*z* equals the molecular ion of ostruthin after the loss of 304 u, the molecular weight of oxypeucedanin hydrate. The loss of 18 u in MS^2^ (603→585) indicates the loss of H_2_O, which could stem from the hydroxylated prenyl side chain of oxypeucedanin hydrate. Fragment *m*/*z* 479 is formed after loss of 124 u, identical to the MS^2^ fragmentation of ostruthin. In MS^3^−18, u (299→281) could be interpreted as a loss of the hydroxy substituent of ostruthin at C7 as H_2_O. Strong fragment ions at *m*/*z* 177 and *m*/*z* 175 indicate major fragments of ostruthin after the loss of the prenyl substituent, with different degrees of hydrogenation. MS^4^ did not provide additional structural information. ^1^H NMR spectra showed two sets of signals that matched with single compounds **1** and **6** (ratio 100:77), but no molecular link could be found (Supplementary Data, Table S2, Figures S1 and S2). Hence, we cannot exclude that in solution under the chromatographic conditions of LC-PDA-MS analysis, either a complex between **1** and **6** or a 2 + 2 cycloaddition product is formed.

Regarding these results, isolated compounds could be identified as follows: oxypeucedanin hydrate (compound **1**), imperatorin (compound **4**), ostruthin (compound **6**), and an unknown compound, tentatively a complex between **1** and **6** (compound **7**).

A correlation was found in the co-occurrence of oxypeucedanin hydrate and the unknown compound (0.974, *p* = 0.002) and oxypeucedanin and imperatorin (0.876, *p* = 0.001) in the fractions of *P. ostruthium* extracts.

### 2.2. Antimicrobial Activity

The antimicrobial (anti-mycobacterial) activity of *P. ostruthium* extracts H and E, their fractions (H1-H5 and E1-E9), and isolated pure compounds oxypeucedanin hydrate (**1**), unknown compound **7** (Ukn7), imperatorin (**4**), and ostruthin (**6**) have been tested against *M. smegmatis* mc^2^ 155 wild type and the LfrA efflux pump-deficient mutant *M. smegmatis* mc^2^ 155 Δ*lfrA*. As controls for comparison of anti-mycobacterial effects, the antimicrobials rifampicin, isoniazid, as well as EtBr were tested (Table 2).

The extracts H and E show the same effect on both the *M. smegmatis* wild type and Δ*lfrA* mutant (MIC of 31.25 mg/L). The different fractions, on the other hand, show different activities. Of the H extract fractions, the H3 fraction had the strongest activity with an MIC of 62.5 mg/L. Although, this was still weaker compared to the original H extract (MIC 31.25 mg/L). Of the E extract fractions, E2, E3, and E4 had the strongest effect, with 4-fold (E2 and E4; MIC 7.8125 mg/L) and 8-fold (E3; MIC 3.91 mg/L) increased anti-mycobacterial activity as compared to the original E extract. Of the four tested pure compounds, **6** showed superior anti-mycobacterial activity (MIC of 1.95 mg/L) compared to the original extracts, fractions, and the tested antibiotics. Ostruthin (**6**) had similar antimicrobial effects to those of the antibiotic isoniazid (MIC 4 mg/L) and better activity than that of rifampicin (MIC 16 mg/L) or EtBr (MIC 8 mg/L).

These results were confirmed by the correlation analysis of the chemical composition and MIC of the fractions. A negative medium correlation between MIC in *M. smegmatis* wild type with imperatorin (**4**) (−0.568, *p* = 0.037) and isoimperatorin (**5**) (−0.595, *p* = 0.028), and a strong negative correlation with ostruthin (**6**) (−0.839, *p* < 0.001) content in the fractions was determined. This indicates a stronger anti-mycobacterial activity of *P. ostruthium* fractions with increased **4** and **6** content leading to the conclusion that compound **6** is, for the most part, responsible for the anti-mycobacterial activity of the *P. ostruthium* extracts, but also that compounds **4** and **5** affect the antimicrobial effect to some extent. On the other hand, oxypeucedanin hydrate and the unknown compound have a medium positive correlation with MIC (0.532, *p* = 0.055 and 0.568, *p* = 0.037), implying their antagonistic action against antimicrobial compounds in *P. ostruthium* extracts.

The susceptibility of the *M. smegmatis* wild type and Δ*lfrA* mutant to the tested samples was similar. Some differences (fold change, FC = 4) were observed in the susceptibility to H2-H4 and E5-E9. The strongest increase in Δ*lfrA* mutant susceptibility, compared to the wild type, was observed for EtBr (Table 2).

These results indicate only a weak involvement of the LfrA efflux pump as a resistance mechanism of *M. smegmatis* against *P. ostruthium* and its coumarins. This was also confirmed by statistical analysis, which shows a strong positive correlation of *P. ostruthium* anti-mycobacterial activity in *M. smegmatis* wild type and Δ*lfrA* mutant (+0.966, *p* < 0.001), indicating a very similar response of both the wild type and mutant strain. A far stronger involvement was observed in the case of EtBr, where the Δ*lfrA* mutant had a 16-fold increase in susceptibility.

### 2.3. Resistance Modulation

The resistance-modulatory activity of *P. ostruthium* extracts H and E in two sub-inhibitory concentrations, 0.25 × MIC and 0.12 × MIC, and isolated pure compounds **1**, **4,** and **6** at 0.25 × MIC were tested in combination with antimicrobials rifampicin, isoniazid, and EtBr. Effective modulation of *M. smegmatis* resistance was set as MF > 2 (Table 3).

No significant resistance-modulatory effect of the tested samples was seen in combination with rifampicin. When tested in combination with isoniazid, a significant effect was observed for the E extract and **4**, with an MF of 8 and 4, respectively. The modulatory effect of tested samples was stronger in combination with EtBr, except for **1**, which showed no effect. Both H and E had an MF 8 at 0.25 × MIC and MF 4 at 0.12 × MIC (Table 3). Ostruthin (**6**) with an MF of 4 had a suitable modulatory effect, although the effect of **4** was far superior with an MF of 16. This strong effect of compound **4** on EtBr susceptibility of *M. smegmatis* indicates that the LfrA efflux pump, responsible for the excretion of EtBr out of the cell, may be one of its main targets. Furthermore, this implies that the modulation of resistance is largely due to efflux pumps inhibition as a mechanism of resistance modification.

### 2.4. Efflux Pump Inhibition

To evaluate the efflux pump inhibitory activity of *P. ostruthium,* extracts and fractions were tested with the EtBr accumulation assay. As a positive control of efflux pumps inhibition, the known EPI verapamil was used. EtBr emits fluorescence once in the bacterial cells bound to DNA, and a stronger fluorescence thus shows a stronger accumulation of EtBr in the cells of *M. smegmatis.* An increase in EtBr accumulation is indicative of a weaker efflux pump activity. When comparing a treated culture with the untreated control, the increase in EtBr accumulation shows efflux pump inhibition by the tested extract or compound.

**Figure 2:**
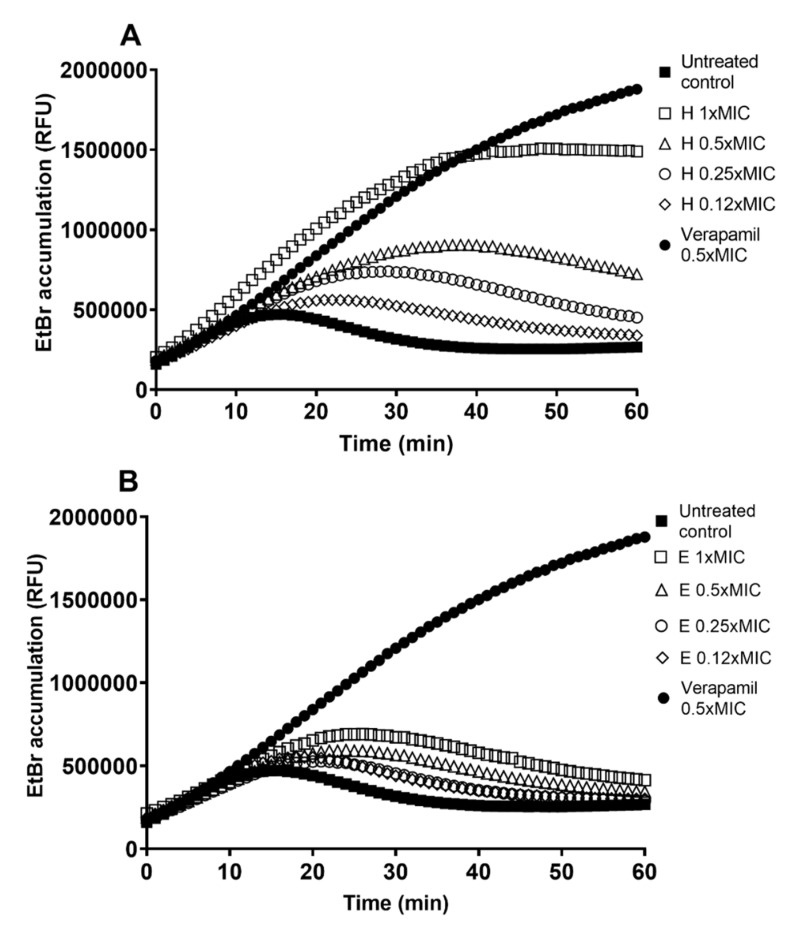
Ethidium bromide (EtBr) accumulation in *M. smegmatis* mc^2^ 155 untreated culture (control) and treated with (**A**) the crude hexane (H) and (**B**) ethanol (E) extract at the minimal inhibitory concentration (MIC; 31.35 mg/L), and sub-inhibitory concentrations of 0.5 × MIC (15.62 mg/L), 0.25 × MIC (7.81 mg/L) and 0.12 × MIC (3.91 mg/L), and the known EPI verapamil in 0.5 × MIC (250 mg/L), presented as relative fluorescent units (RFU). A higher fluorescence indicates stronger efflux pump inhibition.

The extracts H and E were tested in inhibitory concentration (1 × MIC) and sub-inhibitory concentrations of 0.5 × MIC, 0.25 × MIC, and 0.12 × MIC (Figure 2). The efflux pump inhibitory effect of H (Figure 2A) was far superior to the effect of E (Figure 2B). Although E causes some increase in EtBr accumulation (up to a 1.7-fold increase in treated culture vs. untreated control at 60 min treatment time), it is weak compared to the effect of H (up to 5.7-fold increase) and the known EPI verapamil (6.9-fold increase). The effect of extract H is concentration-dependent and strongest at 1 × MIC (Figure 2A).

**Figure 3:**
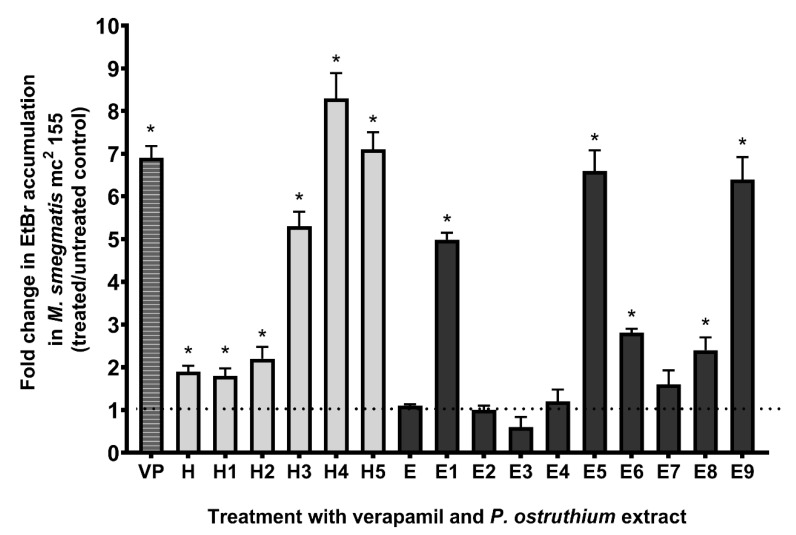
Fold change in ethidium bromide (EtBr) accumulation in *M. smegmatis* mc^2^ 155 culture treated with a sub-inhibitory concentration of 0.25 × MIC of *P. ostruthium* hexane (H) and ethanol (E) extract and their fractions (H1-H5 and E1-E9), and the known EPI verapamil (VP), compared to untreated control at 60 min treatment time point. The dotted line presents no change in EtBr accumulation fold change (baseline). * *p* < 0.05.

Of the tested fractions, H3, H4, H5, E1, E5, and E9 show the strongest increase (>5-fold) in *M. smegmatis* EtBr accumulation (Figure 3). The effect of H4 (8.3-fold increase) surpasses even the effect of the known EPI verapamil.

Correlation analysis of the chemical composition of fractions and EtBr accumulation results have shown a medium positive correlation between the increased presence of **4** (+0.674, *p* = 0.01) and **2** (+0.581, *p* = 0.032), in tested fractions, with an increase in EtBr accumulation. Furthermore, a negative correlation was determined between EtBr accumulation and content of **1** (−0.682, *p* = 0.006) and the unknown compound **7** (−0.651, *p* = 0.01). This indicates that a higher content of these two compounds weakens efflux pump inhibition of *P. ostruthium* extracts.

### 2.5. Effect of Imperatorin and Ostruthin on EtBr Accumulation in M. smegmatis mc^2^ 155 Wild Type and ΔlfrA Mutant

**Figure 4:**
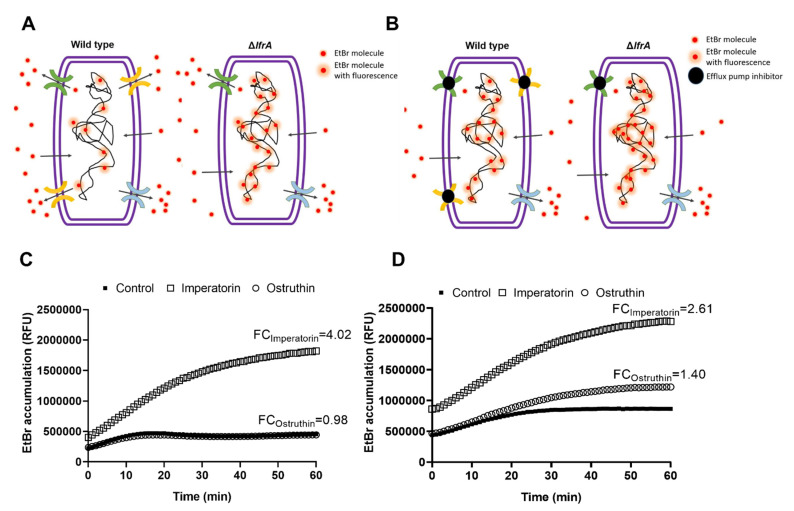
Schematic representation of ethidium bromide (EtBr) accumulation and efflux in *M. smegmatis* mc^2^ 155 wild type and Δ*lfrA* mutant without inhibitor (**A**) and with efflux pumps inhibitor (**B**); and EtBr accumulation in *M. smegmatis* mc^2^ 155 (**C**) and *M. smegmatis* mc^2^ 155Δ*lfrA* (**D**) untreated culture (control) and treated with the isolated compounds imperatorin (**4**) and ostruthin (**6**) at the sub-inhibitory concentration of 0.25 × MIC, presented as relative fluorescent units (RFU) with indicated FC in EtBr accumulation in the treated culture vs. the untreated control.

The results so far indicate that **4** and **6** play a vital role in *P. ostruthium* anti-mycobacterial effect. Furthermore, **4** has been implicated as a crucial player in the resistance-modulatory effect and efflux pumps inhibition, with one of its main targets being the LfrA efflux pump. To confirm this observation, we tested the EtBr accumulation of both *M. smegmatis* mc^2^ 155 wild type and the Δ*lfrA* mutant treated with **4** and **6** at the sub-inhibitory concentration 0.25 × MIC. Efflux pumps are responsible for excreting EtBr from the bacterial cell as it is a toxic compound when accumulated (Figure 4A). Bound to DNA, EtBr emits fluorescence, which can be detected. As the *M. smegmatis* mc^2^ 155Δ*lfrA* mutant has no LfrA efflux pump, more bound EtBr is to be expected and thus more fluorescence emitted, compared to the wild type (Figure 4A). Although, the mutant strain still has other efflux systems. Efflux pump inhibitors block the action of efflux pumps and cause the increase in EtBr accumulation in cells treated with an inhibitor (Figure 4B), compared to the untreated control.

Imperatorin (**4**) and ostruthin (**6**) caused an EtBr accumulation of 4.02- and 0.98-fold, respectively, in *M. smegmatis* wild type (Figure 4C), and 2.61- and 1.40-fold, respectively, in the Δ*lfrA* mutant (Figure 4D). The lower effect of imperatorin on EtBr accumulation of the Δ*lfrA* mutant compared to the wild type confirms the LfrA efflux pump as one of the major targets of imperatorin action, although not the only one. Although weaker, a still significant increase in EtBr accumulation in the Δ*lfrA* mutant treated with imperatorin, was observed. Ostruthin, on the other hand, had no effect on *M. smegmatis* wild type but showed an increase in Δ*lfrA* mutant EtBr accumulation.

### 2.6. Effect of Extracts, Imperatorin and Ostruthin on Membrane Permeabilization in M. smegmatis mc^2^ 155 Wild Type and ΔlfrA Mutant

**Figure 5:**
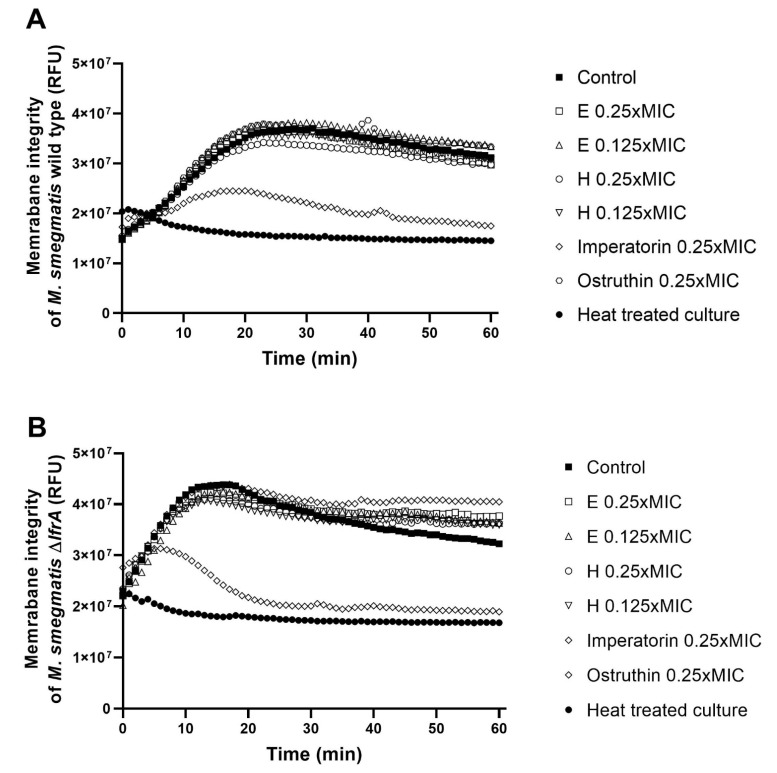
Membrane integrity of M. smegmatis wild type (**A**) and the ∆lfrA mutant (**B**) in untreated control culture and culture treated with ethanol extract at concentrations of 0.25 and 0.125 × MIC (E 0.25 × MIC and E 0.125 × MIC), hexane extract at 0.25 and 0.125 × MIC (H 0.25 × MIC and H 0.125 × MIC), imperatorin at 0.25 × MIC, and ostruthin at 0.25 × MIC. Heat-treated culture was used as a control for total membrane disruption. Membrane integrity is presented as relative fluorescent units (RFU) through a 60 min treatment time.

In order to evaluate the impact of hexane and ethanolic extracts as well as imperatorin (**4**) and ostruthin (**6**) on membrane permeabilization, at sub-inhibitory concentrations, fluorescence quenching of SYTO9 by propidium iodide was measured. Only imperatorin presented a significant (*p* < 0.01) disruption of the membrane of both tested strains. The disruptive effect was more prominent in the mutant strain (86%) as compared to the wild type (76%) (Figure 5, Table S3).

## 3. Discussion

Especially in times of rising bacterial resistance, it is important to consider alternative and adjuvant therapy approaches for serious infections such as tuberculosis. In this study, we used *M. smegmatis* as a model organism to evaluate the anti-mycobacterial profile of *P. ostruthium* hexane and ethanol extracts, subsequent fractions, and isolated coumarins and to investigate its resistance modifying and EPI activity in mycobacteria.

The chemical characterization of *P. ostruthium* extracts and fractions and the subsequent isolation of pure coumarins allowed us a more in-depth analysis of the antimicrobial, resistance-modulatory, and efflux pump inhibitory potential of this plant. Referencing Hadaček et al. [28] and Vogl et al. [19], we also identified six major coumarins in *P. ostruthium* extracts, namely oxypeucedanin hydrate (**1**), oxypeucedanin (**2**), ostruthol (**3**), imperatorin (**4**), isoimperatorin (**5**), and ostruthin (**6**). Furthermore, four identified compounds **1**, **4**, **6,** and an unknown compound **7**, tentatively a complex between **1** and **6** (Ukn7), were isolated and tested.

The antimicrobial activity of both extract H and E of *P. ostruthium* was similar, as seen in the MIC assay. Interestingly, most of the tested H fractions showed weaker activity than the main extract H, even though they had higher concentrations of certain pure compounds (e.g., isoimperatorin (**5**) in H2 and H3, and imperatorin (**4**) in H5). This is not surprising as we do know that in some combinations, the synergistic activity can generate greater effects than some single pure compounds alone [29,30]. Furthermore, in these extracts, we found antagonistic effects of oxypeucedanin hydrate (**1**) and the unknown compound **7** regarding their antimicrobial activity, which explains the seeming discrepancy in MIC. In general, plant extracts are a mixture of many compounds with warring activities, with both synergistic and antagonistic effects. This makes the identification of pure active compounds or a combination of these an important task for finding effective plant-derived antimicrobials. However, we found that the antimicrobial activity of *P. ostruthium* can be mainly linked to its ostruthin (**6**) content, although **4** and **5** may also affect the antimicrobial efficiency to some extent. We particularly observed that those *P. ostruthium* extracts and fractions containing high amounts of ostruthin (**6**) exhibited stronger antimicrobial effects.

The antimicrobial activity results of pure compounds **1**, **4** and **6**, suggest that the more lipophilic the substrate, the greater the antimicrobial effect. This can be deduced from calculated logP values (**1**: 1.575; **4**: 2.983; **6**: 5.295 [31]). Of all tested compounds, **6** induced the most potent activity, causing even greater effects than those of rifampicin and isoniazid, the two frontline anti-tuberculotic drugs. In a study by Schinkovitz et al. [26], the antimicrobial potential of **6** was first reported, and this strong anti-mycobacterial activity was also confirmed in this study. Furthermore, our results of the antimicrobial activity of **1** and **4** are comparable to the results of Widelski et al. [14], where they tested isolated coumarins against Gram-positive and Gram-negative bacteria and found MICs of 12–70 mg/L for **4**, but 10-times higher MICs (550–800 mg/L) for **1**. This implies that these coumarins have a general mode of action targeting both Gram-positive and Gram-negative bacteria and could have a wider use as adjuvant antimicrobials.

The deletion of the *lfrA* gene has only weak involvement in the resistance of *M. smegmatis* against *P. ostruthium* and its isolated coumarins, as it did not render the mutant strain more susceptible to the substrates, including the antimicrobials rifampicin and isoniazid. However, it did affect *M. smegmatis* susceptibility toward EtBr (16-fold decrease in MIC), which is in agreement with the study of Li et al. [4]. They showed that the deletion of the *lfrA* gene leads to two- to eight-fold decreases in the MICs of multiple drugs such as fluoroquinolones, ethidium bromide, and acriflavine. LfrA *of M. smegmatis* belongs to the MFS transporters [11] and is generally known to confer low-level resistance to fluoroquinolones, as well as some other toxic compounds such as EtBr [4], but according to our results, not to coumarins.

In antimicrobial resistance modulation, we observed only a marginal effect of *P. ostruthium* extracts and its coumarins on the antimicrobial activity of rifampicin or isoniazid, but a strong effect of imperatorin (**4**) combined with EtBr, leading to a 16-fold higher potency. This striking modulating effect of compound **4** on the EtBr susceptibility of *M. smegmatis* would indicate the LfrA efflux pump, responsible for the excretion of EtBr out of the cell, as being one of the main targets. These results show that ostruthin has a comparable resistance-modulatory activity to other well-known EPIs such as verapamil and reserpine and plant-derived EPIs [29,32], but the activity of **4** surpasses even that.

The strong resistance modulating potential of compound **4** was confirmed in the following EtBr accumulation experiments. Imperatorin (**4**) was discovered as a strong efflux pump inhibitor, with the LfrA efflux pump as its main target. In the EtBr accumulation assay, the lower effect of **4** on EtBr accumulation in the Δ*lfrA* mutant compared to the wild type presents the LfrA efflux pump as one of the major targets of **4**. Yet, it has to be taken into account that the membrane permeabilization by compound **4** at 0.25 MIC could additionally increase EtBr accumulation and overlap with observed efflux inhibition. However, we do not conclude that the pronounced EtBr accumulation is only due to increased membrane permeability, as we see a stronger EtBr accumulation by **4** in the wild-type strain than in the *lfrA* mutant, although membrane permeabilization was higher in the latter.

Although weaker, a still significant increase in EtBr accumulation in the Δ*lfrA* mutant was observed when treated with compound **4**, which also suggests other efflux pumps as targets for imperatorin (**4**) action; hence, no exclusive selectivity of **4** for LfrA was recognized.

Ostruthin (**6**), on the other hand, had no effect on *M. smegmatis* wild type but could enhance the accumulation of EtBr in the Δ*lfrA* mutant. One reason for this phenomenon might be the expression of other usually scarce efflux pumps, which operate in the absence of the LfrA efflux pump. Such other efflux pumps could be, for example, the homologs of *Mycobacterium tuberculosis* Rv1145, Rv1146, Rv1877, Rv2846c (*efpA*), and Rv3065 (*mmr* and *emrE*) [4]. In the study by De Siena et al. [33], two other ortholog operons, Rv1687/86/85c in *M. tuberculosis* and MSMEG_3762/63/65 in *M. smegmatis*, both designated as ABC efflux pump systems, were identified, sharing a high percentage of identity. It was shown that the protein complex MSMEG-3762/63, annotated as an efflux pump, is involved in the resistance toward first- and second-line anti-TB drugs, such as rifampicin and ciprofloxacin, as well as in the formation of mycobacterial biofilms. Furthermore, Cossu et al. [34] observed that MSMEG_3765 and its ortholog Rv1685c in *M. tuberculosis*, which code for TetR-like regulator family, were upregulated in acid-nitrosative stress conditions. Especially members of the TetR family of transcriptional regulators (TFTRs) are present in a large number of bacterial genomes, regulating a wide range of cellular functions, including efflux pump activity. For instance, TFTRs were found to be the most abundant family of transcriptional regulators, with 906 TFTRs identified in 10 mycobacterial species, including *M. smegmatis* [35,36].

In this study, we used the mutant strain Δ*lfrA* to characterize the relevance of LfrA in mycobacterial resistance. The role of this efflux pump was confirmed by the EtBr accumulation assay, where loss/absence of LfrA resulted in greater accumulation of EtBr in the mycobacterial cell. While the inhibition of efflux activity of LfrA can be proposed as one plausible mechanism for the antimicrobial efficacy of compounds **4** and **6** against *M. smegmatis*, there are likely other efflux systems such as TFTRs and/or ABC-transporters (e.g., MSMEG_3762/63/65) involved. However, this needs to be confirmed in further experiments.

There has been much research performed on genes involved in the intrinsic and acquired antimicrobial drug resistance of mycobacteria, including MDR/XDR *M. tuberculosis.* Besides the role of efflux pumps in antibiotic resistance, they might also be involved in bacterial behaviors associated with virulence, biofilm development, and quorum sensing-dependent expression of virulence factors [33,37,38]. Based on the findings of this study, the interplay of **4** and **6** in *P. ostruthium* fractions seems to be crucial for efflux pump inhibitory-, antibacterial, and resistance-modulatory effects of this plant. While imperatorin (**4**) may have a higher impact on efflux pump inhibition in *M. smegmatis,* ostruthin (**6**) turned out to be a strong antimicrobial agent. Especially, the combined effect of both compounds **4** and **6** in *P. ostruthium* may lead to greater outcomes in antimicrobial efficacy.

Due to these findings, *P. ostruthium* can be considered a suitable candidate for adjuvant therapeutic approaches to mycobacterial infections.

## 4. Materials and Methods

### 4.1. Plant Extract Preparation

*P. ostruthium* (L.) Koch (masterwort) roots were supplied as dry pre-cut material by Kottas pharma (Vienna, Austria), a voucher sample is stored at the herbarium of the Department of Pharmacognosy. Before extract preparation, the material was ground.

The *P. ostruthium* hexane extract (H) was prepared from 202.00 g of plant material by adding 1.2 L of hexane (Roth, Germany) and mixing with a magnetic stirrer for 24 h and filtered (Rotilabo pleated paper filters; Roth, Germany). The retentate was subjected to further extraction with 600 mL of hexane for 72 h and subsequent filtration. The filtrates were evaporated with a rotary evaporator (Rotavapor; Büchi, Switzerland) under vacuum at 35 °C. The yield of the hexane extraction was 5.21 g of H.

The *P. ostruthium* ethanol extract (E) was prepared by ethanol Soxhlet extraction by adding 1.5 L of 96% denatured ethanol (Roth, Germany) to 190.02 g of plant material placed into a cellulose thimble and the Soxhlet extractor. The extraction was performed for 3 h at 200 °C. The solvent was evaporated with a rotary evaporator at 50 °C followed by drying with nitrogen flow. The yield of the ethanol extraction was 20.43 g of E.

### 4.2. Fractionation and Isolation

Fractions of crude extracts H and E were obtained through vacuum liquid chromatography (VLC) on silica gel (0.040–0.063 mm, Merck, Darmstadt, Germany) with solvent mixtures of increasing polarity (hexane, ethyl acetate, methanol, all Roth, Germany). Fractions were controlled by thin-layer chromatography (silica gel 60 F_254_, Merck, Darmstadt, Germany) and those of similar substance patterns combined to yield five fractions for H (H1–H5) and 9 fractions for E (E1–E9).

Four compounds were isolated by semi-preparative HPLC using a Shimadzu CBM-20A controller, LC-20AT solvent delivery module, SIL-10AF autosampler, CTO-20AC column oven, SPD-M20A diode array detector, and FRC-10A fraction collector (all Shimadzu, Kyoto, Japan).

All separations were performed on a Luna C18(2) column, 250 × 10 mm, 10 μm (Phenomenex, Torrance, CA, USA) via isocratic elution with a flow rate of 4 mL/min, 25 °C column temperature; 200 μL injection volume.

Compounds **1** and **7** were obtained from E8 (VLC fraction with hexane:ethyl acetate:methanol = 4:80:16) by isocratic elution with a solvent composition of acetonitrile:water = 70:30 (*v*/*v*). Compound **4** was isolated with acetonitrile:water = 63:37 (*v*/*v*) from H4 (hexane:ethyl acetate:methanol = 36:60:4) and compound **6** with acetonitrile:water = 75:25 (*v*/*v*) from E2 (hexane:ethyl acetate = 70:30).

### 4.3. Chemical Analysis

LC-PDA-ESI-MS^n^ was used to analyse extracts, subfractions, and isolated compounds using a Dionex Ultimate 3000 RS LC system in combination with an LTQ XL linear ion-trap mass spectrometer and ESI ion source (Thermo Scientific, Waltham, MA, USA). Separation was carried out on a Zorbax SB-C18 RapidResolution HD column with dimensions of 100 × 2.1 mm and 1.8 µm particle size (Agilent, Santa Clara, CA, USA). Solvents for gradient elution were 0.1% formic acid in water (A) and acetonitrile (B). Elution started with 40% B, rising to 100% B between 0 and 11.11 min, falling from 100% B to 40% B from 11.11 to 11.48 min, and equilibrating until 15 min at a flow rate of 0.39 mL/min. The column temperature was kept at 35 °C, injection volume for samples was 2 µL. Absorbance was detected in a wavelength range of 190 nm to 500 nm using a PDA detector. Mass spectral detection was carried out in positive ion mode in *m*/*z* ranges of 50 to 2000 amu. MS conditions were set to a source voltage of 3.5 kV, capillary temperature of 300 °C, source temperature of 350 °C, sheath gas flow of 65 arb (arbitrary units), and auxiliary gas flow of 15 arb.

The relative composition of identified coumarins in subfractions in percent was calculated from peak areas resulting from PDA-UV chromatograms at a wavelength of 310 nm.

### 4.4. Bacterial Strains and Culture Conditions

*M. smegmatis* mc^2^ 155 wild-type strain (ATCC 700084, LCG Promochem, Teddington, Middlesex, UK) and mutant strain *M. smegmatis* mc^2^ 155Δ*lfrA* (kindly provided by Prof. José A. Aínsa, Departamento de Microbiología, Facultad de Medicina, Universidad de Zaragoza, Spain) were inoculated onto Columbia blood agar (Oxoid, Hampshire, UK) supplemented with 5% defibrinated horse blood (Oxoid, Hampshire, UK), and grown for 72 h at 37 °C in aerobic conditions.

*M. smegmatis* mc^2^ 155Δ*lfrA* represents a mutant strain lacking the *lfrA* gene encoding the LfrA efflux pump [12]. Bacterial cultures were stored with the Viabank system (mwe medical wire, Witshire, UK) at −80 °C.

### 4.5. Antimicrobial Activity Testing

The antimicrobial activity of *P. ostruthium* extracts H and E, their fractions, and isolated pure compound **1**, **4**, **6**, and the unknown compound **7** as well as the antimicrobials rifampicin (Sigma-Aldrich, Poole, UK), isoniazid (Sigma-Aldrich, Poole, UK), and ethidium bromide (Sigma-Aldrich, Poole, UK) were determined using the broth microdilution assay as described before [39], with some modifications. Briefly, *P. ostruthium* extracts, fractions, and pure compound were prepared in dimethyl sulfoxide (DMSO ≥ 99.5%, Roth, Germany) at 40,000 mg/L and further diluted in Mueller Hinton broth (MHB; Oxoid, Hampshire, UK) to a final concentration of 2000 mg/L and 5% DMSO. Isoniazid was prepared at a final concentration of 32 mg/L and rifampicin at 256 mg/L in MHB with 5% DMSO. EtBr was prepared to a final concentration of 64 mg/L in MHB. Two-fold serial dilutions of the tested substances were prepared in 96-well microtiter plates (TPP, Merck, Darmstadt, Germany) to a final volume of 100 μL. A bacterial solution was prepared in MHB to an optical density (OD_600_) of 0.23 and further diluted to reach 5 × 10^5^ CFU/mL. The bacterial solution was added to the wells of the microtiter plates to a final volume of 200 μL. Plates were incubated at 37 °C in aerobic conditions for 72 h. The minimal inhibitory concentration (MIC) was determined by the addition of 20 μL of thiazolyl blue tetrazolium bromide (MTT, 20 mg/mL in methanol, Sigma-Aldrich, Vienna, Austria). The MIC was set as the lowest concentration of a tested sample, which still inhibited the growth of *M. smegmatis* wild type and Δ*lfrA* mutant.

### 4.6. Modulation Factor Analysis

The resistance-modulatory activity was determined for *P. ostruthium* extracts H and E at 0.25 × MIC and 0.12 × MIC, and pure compounds **1**, **4**, and **6** at 0.25 × MIC according to the method described by Gröblacher et al. [39], with few modifications. Briefly, the tested modulators were prepared in MHB and 1% DMSO at 2 × final testing concentration. The antimicrobials rifampicin, isoniazid, and EtBr were two-fold diluted in the prepared modulator solution and in fresh MHB, in a 96-well plate, to a final volume of 100 μL. A bacterial solution (5 × 10^5^ CFU/mL, described above) was added to each well to the final volume of 200 μL. The plates were incubated at 37 °C in aerobic conditions for 72 h. The MIC of tested antimicrobials was determined without the modulator (MHB) and with the modulator, as described above. For the determination of resistance modulation of the tested samples, the modulation factor (MF) was calculated according to the following formula. The significance of resistance modulation was set at MF > 2.

MF = (MIC antibiotic)/ (MIC antibiotic + modulator) (1)

### 4.7. Ethidium Bromide Accumulation Assay

To determine the efflux pump inhibitory activity of *P. ostruthium* extracts, fractions, and pure compounds in *M. smegmatis*, the EtBr accumulation assay was performed following the protocol of Gröblacher et al. [39], including modifications.

EPI solution preparation: A solution consisting of 250 μL 1.6% glucose (Sigma-Aldrich, Vienna, Austria), 25 μL EtBr (20 mg/L), and 200 μL sterile distilled water was prepared. To this solution, an EPI in a 40 × higher concentration than the desired testing concentration in DMSO or only DMSO with 0.05% tween (untreated control) was added. The known EPI verapamil was used as positive control at 0.5 × MIC (256 mg/L). Culture preparation: The *M. smegmatis* culture was inoculated into Middlebrook 7H9 broth supplemented with the Middlebrook ADC supplement (Difco, Becton, Dickinson and Company, England, UK) and 0.05% Tween 80 (Sigma-Aldrich, Vienna, Austria) and incubated overnight (16 h) at 37 °C, 180 rpm, in aerobic conditions. After incubation, the culture was centrifuged (4000 rpm, 5 min, 20 °C) and washed with PBS supplemented with 0.05% Tween 80. The culture OD_600_ was adjusted to 0.4. The culture and EPI solution were mixed at a 1:1 (*v*/*v*) ratio and added to a black microtiter plate (Nunc, Thermo Scientific, Denmark) at a final volume of 200 μL. An EPI solution mixed with PBS with 0.05% Tween 80 was prepared for the blank value. For imperatorin and ostruthin, and no fluorescence emission interference under the test conditions could be recognized (data not shown). This is also in agreement with the study of Frerot and Decorzant [40], who recorded much lower excitation and emission wavelengths for a number of furanocoumarins. The fluorescence of *M. smegmatis* culture accumulating EtBr was measured with a microplate reader (Hidex Sense Microplate Reader, HVD GmbH, Austria) with parameters set at 531 nm excitation and 590 emission wavelengths at 37 °C in one-minute intervals for one hour. The results are presented as relative fluorescent units (RFU) or as fold increase in EtBr accumulation in the treated culture compared to the untreated control at the 60 min treatment time point.

### 4.8. Membrane Integrity Assay

Effects on membrane integrity of *M. smegmatis* strains were tested for imperatorin (**4**), ostruthin (**6**), hexane, and ethanol extracts at sub-inhibitory concentrations using Live/Dead bacterial viability kits (L-7012; Molecular Probes, Eugene, OR, USA) in the membrane disruption assay as according to Šimunović et al. [30]. *M. smegmatis* culture was prepared as described in 4.7. Compounds were prepared in 50 × higher concentrations than desired test concentrations in DMSO and added to a culture of OD600 = 0.4. The Live/Dead solution was prepared in 0.8% glucose solution according to manufacturer instructions and added to the culture at 1:1 (*v*/*v*) in black microtiter plates (Thermo Fisher Scientific, Roskilde, Roskilde, Denmark) at a final volume of 200 µL. A heat-treated culture (80 °C, 30 min) served as a negative control, an untreated culture as a positive control. SYTO9 fluorescence was measured using a microplate reader (Hidex Sense Microplate Reader, HVD GmbH, Austria) at λex = 485 nm and λex = 535 nm in one-minute intervals over one hour, and measurements were performed in triplicate.

The membrane disruption was calculated as:

Membrane disruption (%) = 100 − (Control culture (RFU)-Heat-treated culture (RFU))/(Treated culture (RFU)-Heat-treated culture(RFU))*100 (2)

## Figures and Tables

**Table 1:** MS^n^ Fragmentation and UV maxima of main coumarins identified in *Peucedanum ostruthium* extracts and subsequent fractions, * relative fragment intensities in MS^n^ > 10%.

Nr.	Identification	[M + H]^+^, *m*/*z*	MS^n^ *, *m*/*z*	λ_max,_ nm
**1**	Oxypeucedanin hydrate	305	MS^2^ (305): 203 MS^3^ (203): 159, 147, 175, 131 MS^4^ (159): 131	221, 250, 259, 266, 311
**2**	Oxypeucedanin	287	MS^2^ (287): 203 MS^3^ (203): 159, 147, 175, 131 MS^4^ (159): 131	220, 250, 265sh, 308
**3**	Ostruthol	387	MS^2^ (387): 369, 185, 305, 167 MS^3^ (369): 167, 287, 269, 203 MS^4^ (167): 83	220, 249, 267, 310
**4**	Imperatorin	271	MS^2^ (203): 175, 147, 159, 131 MS^3^ (175): 147 MS^4^ (147): 119, 147, 91	218, 249, 265sh, 301
**5**	Isoimperatorin	271	MS^2^ (203): 159, 147, 175, 131 MS^3^ (159): 131 MS^4^ (131): 103, 131	221, 250, 265sh, 309
**6**	Ostruthin	299	MS^2^ (299): 175 MS^3^ (175): 147 MS^4^ (147): 119	225, 247, 256sh 295sh, 331
**7**	Unknown compound 7	603	MS^2^ (603): 299, 585, 479, 401, 383, 461 MS^3^ (299): 281, 177, 175, 189, 243, 203, 217 MS^4^ (281): 225, 239, 263, 211, 253, 252, 199, 266, 185, 237, 238, 187, 161	219, 249, 267sh, 301, 335sh

**Table 2:** Antimicrobial activity of *P. ostruthium* crude hexane (H) and ethanol extract (**E**) and their fractions (H1–H5 and E1–E9), pure compounds oxypeucedanin hydrate (**1**), Ukn7 (**7**), imperatorin (**4**), and ostruthin (**6**), isolated from the extracts, and antimicrobials rifampicin, isoniazid, and ethidium bromide (EtBr), against *M. smegmatis* mc^2^ 155 wild type and the efflux pump mutant strain Δ*lfrA*, presented as the minimal inhibitory concentration (MIC) in mg/L with the corresponding fold change (FC) in antimicrobial susceptibility of the Δ*lfrA* mutant compared to the wild type.

	*M. smegmatis* mc^2^ 155 Wild Type	*M. smegmatis* mc^2^ 155 Δ*lfrA*	
Substrate	MIC (mg/L)		FC ^a^
H	31.25	31.25	1
H1	>1000	>1000	1
H2	250	62.5	4
H3	62.5	15.625	4
H4	125	31.25	4
H5	250	125	2
E	31.25	31.25	1
E1	250	250	1
E2	7.8125	7.8125	1
E3	3.91	3.91	1
E4	7.81	3.91	2
E5	250	62.5	4
E6	>1000	500	4
E7	>1000	500	4
E8	1000	250	4
E9	>1000	500	4
Oxypeucedanin hydrate (**1**)	250	250	1
Ukn7 (**7**)	62.5	62.5	1
Imperatorin (**4**)	62.5	62.5	1
Ostruthin (**6**)	1.95	1.95	1
Rifampicin	16	16	1
EtBr	8	0.5	16
Isoniazid	4	4	1

^a^ FC—fold change.

**Table 3:** Minimal inhibitory concentration (MIC, mg/L) of rifampicin, isoniazid, and ethidium bromide (EtBr) alone, and in the presence of *P. ostruthium* hexane (H) and ethanolic (E) extracts at 0.25 × MIC (7.82 mg/L) and 0.125 × MIC (3.91 mg/L), and coumarins oxypeucedanin hydrate (**1**), unknown compound **7** (Ukn7), imperatorin (**4**), and ostruthin (**6**) at 0.25 × MIC, as resistance modulators of *M. smegmatis* mc^2^ 155 with the corresponding resistance modulation factor (MF) showing an increase in antibiotic activity in the presence of modulator.

	MIC (mg/L)					
	Rifampicin	MF ^a^	Isoniazid	MF ^a^	EtBr	MF ^a^
Control	16	/	4	/	8	/
H 0.25 × MIC	8	2	4	1	1	8
H 0.12 × MIC	8	2	4	1	2	4
E 0.25 × MIC	8	2	0.5	8	1	8
E 0.12 × MIC	8	2	1	4	2	4
Oxypeucedanin hydrate (**1**) 0.25 × MIC	16	1	4	1	8	1
Ukn7 (**7**) 0.25 × MIC	8	2	4	1	4	2
Imperatorin (**4**) 0.25 × MIC	8	2	1	4	0.5	16
Ostruthin (**6**) 0.25 × MIC	8	2	4	1	2	4

^a^ Modulation factor calculated as a difference between MIC of antibiotics alone and in combination with resistance modulator.

## Data Availability

The data presented in this study are available on request from the corresponding author.

## Supplementary Materials

The following are available online at https://www.mdpi.com/article/10.3390/antibiotics10091075/s1, Figure S1: Oxypeucedanin hydrate (**1**), Figure S2. Ostruthin (**6**), Table S1: NMR Spectroscopic Data (700 MHz, CDCl3) for compound **1**, 25 °C, TMS was used as internal standard, Table S2: NMR Spectroscopic Data (700 MHz, CDCl3) for compound **6**, 25 °C, TMS was used as internal standard, Table S3: Membrane integrity disruption (%) in *M. smegmatis* mc2 155 wild type and ∆*lfrA* mutant after treatment with ethanol extract at concentrations of 0.25 and 0.125 × MIC (E 0.25 × MIC and E 0.125 × MIC), hexane extract at 0.25 and 0.125 × MIC (H 0.25 × MIC and H 0.125 × MIC), imperatorin at 0.25 × MIC, and ostruthin at 0.25 × MIC.
