# Supplementary material for: Efflux Pump Inhibition and Resistance Modulation in Mycobacterium smegmatis by Peucedanum ostruthium and Its Coumarins
Source: Antibiotics (Basel). 2021 Sep 5;10(9):1075. doi: 10.3390/antibiotics10091075 (PMC8472667; doi:10.3390/antibiotics10091075)
Supplement: Supplementary file 1 [file antibiotics-10-01075-s001.zip › antibiotics-1298922-supplementary.pdf]

Supplementary Material

Efflux pump inhibition and resistance modulation in *Mycobacterium smegmatis* by *Peucedanum ostruthium* and its coumarins

Katarina Šimunović, Julia Solnier, Fabian Alperth, Olaf Kunert, Sonja Smole Možina, Franz Bucar

Table S1: NMR Spectroscopic Data (700 MHz, $CDCl_3$) for compound **1,** 25 °C, TMS was used as internal standard.

compound **1**			compound **1**		
position	δ_C, type	δ_H (*J* in Hz)	position	δ_C, type	δ_H (*J* in Hz)
2	161.0, C	-	8a	152.6, C	-
3	113.3, CH	6.32, d (9.7)	9	95.1, CH	7.21, s
4	139.0, CH	8.18, d (9.7)	9a	152.6, C	-
4a	107.4, C	-	1′	74.5, CH_2	4.45, dd (9.8, 7.7)<br>4.54, dd (9.8, 2.8)
5	148.4, C	-	2′	76.5, CH	3.91, dd (7.7, 2.7)
5a	114.4, C	-	3′	71.7, C	-
6	104.8, CH	6.99, d (2.3)	4′	25.2, CH_3	1.32, s
7	145.4, CH	7.62, d (2.3)	5′	26.7, CH_3	1.37, s

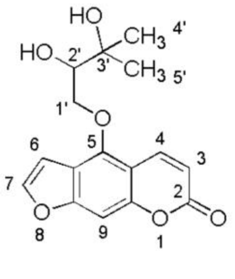

Figure S1. Oxypeucedanin hydrate (**1**)

Table S2: NMR Spectroscopic Data (700 MHz, $CDCl_3$) for compound **6**, 25 °C, TMS was used as internal standard.

compound **6**			compound **6**		
position	δ_C, type	δ_H (*J* in Hz)	position	δ_C, type	δ_H (*J* in Hz)
2	161.4, C	-	1′	29.2, CH_2	3.40, d (7.2)
3	113.2, CH	6.23, d (9.5)	2′	120.6, CH	5.30, m
4	143.5, CH	7.60, d (9.5)	3′	139.9, C	-
4a	112.7, C	-	4′	39.7, CH_2	2.11, m
5	128.4, CH	7.19, s	5′	26.3, CH_2	2.12, m
6	124.6, C	-	6′	123.7, CH	5.06, m
7	158.1, C	-	7′	132.2, C	-
8	104.7, CH	6.78, s	8′	25.8, CH_3	1.68, s
8a	154.4, C	-	9′	17.7, CH_3	1.60, s
7-OH	-	7.60, s	10′	16.3, CH_3	1.77, s

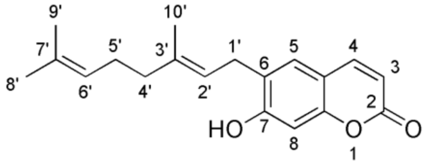

Figure S2. Ostruthin (**6**)

Table S3: Membrane integrity disruption (%) in *M. smegmatis* mc[2] 155 wild type and *ΔlfrA* mutant after treatment with ethanol extract at concentrations of 0.25 and 0.125 x MIC (E 0.25 x MIC and E 0.125 x MIC), hexane extract at 0.25 and 0.125 x MIC (H 0.25 x MIC and H 0.125 x MIC), imperatorin at 0.25 x MIC, and ostruthin at 0.25 x MIC.

	Membrane integrity disruption (%) in *M. smegmatis* mc[2] 155	
Treatment	wild type	Δ*lfrA*
E 0.25xMIC	-14.3 ± 3.5	-29.9 ± 7.4
E 0.125xMIC	-38.2 ± 3.6	-23.5 ± 6.6
H 0.25xMIC	-11.4 ± 6.0	-19.8 ± 6.3
H 0.125xMIC	-20.5 ± 3.1	-19.9 ± 3.3
Imperatorin 0.25xMIC	**76.0 ± 4.1**	**86.1 ± 2.3**
Ostruthin 0.25xMIC	-36.4 ± 1.4	-46.9 ± 5.3
